# The jet mass distribution after Soft Drop

**DOI:** 10.1140/epjc/s10052-018-5579-5

**Published:** 2018-02-02

**Authors:** Simone Marzani, Lais Schunk, Gregory Soyez

**Affiliations:** 1 Dipartimento di Fisica, Università di Genova and INFN, Sezione di Genova, Via Dodecaneso 33, 16146 Genoa, Italy; 2 Deutsches Elektronen-Synchroton DESY, Notkestraße 85, 22607 Hamburg, Germany; 3 IPhT, CEA Saclay, CNRS UMR 3681, 91191 Gif-Sur-Yvette, France

## Abstract

We present a first-principle computation of the mass distribution of jets which have undergone the grooming procedure known as Soft Drop. This calculation includes the resummation of the large logarithms of the jet mass over its transverse momentum, up to next-to-logarithmic accuracy, matched to exact fixed-order results at next-to-leading order. We also include non-perturbative corrections obtained from Monte-Carlo simulations and discuss analytic expressions for hadronisation and Underlying Event effects.

*Introduction* The study of jets at the Large Hadron Collider (LHC) has recently taken a new turn with new substructure observables [1, 2] amenable to precise theory calculations [3–5], including genuine theory uncertainty bands, and corresponding experimental measurements from both the CMS [6] and ATLAS [7] collaborations. The substructure techniques we concentrate on are usually referred to as *grooming* and they aim to reduce sensitivity to non-perturbative corrections and pileup.

A first series of studies has focused on the jet mass after applying the (modified) MassDrop Tagger (mMDT) [1, 8] in dijet events, as measured by the CMS collaboration [6]. On the theory side, the description of this observable requires to match a resummed calculation, important in the small-mass region, to fixed-order results, which are important for large masses. The former are obtained analytically, including to all orders terms enhanced by the large logarithms of $p_t^2/m^2$ with $p_t$ the jet transverse momentum and *m* the (groomed) jet mass. The latter is obtained from fixed-order Monte-Carlo simulations. To date, two theory calculations are available: a SCET-based next-to-leading logarithmic (NLL) resummation in the small $z_{\text {cut}}$ limit, matched to leading order (LO) results [4], and our previous study matching a leading logarithmic resummation, including finite (but small) $z_{\text {cut}}$ effects, to next-to-leading order results [5]. Comparing both predictions, we see a small NLL effect at small mass and non-negligible NLO corrections at large mass.

The goal of the present letter is to extend our mMDT study from Ref. [5] to the case of Soft Drop  [2], i.e. allowing for a non-zero value of the angular exponent $\beta$. When $\beta \ne 0$, the logarithmic counting differs from the mMDT case, essentially because Soft Drop retains soft-collinear radiation, which is always groomed away by mMDT. In this case, the SCET-based calculation from Ref. [4] reaches NNLL accuracy and it is matched, in the dijet case, to LO fixed-order results. Here, we present the results of a NLL resummation matched to NLO fixed-order accuracy.^1^

After a brief review of the Soft Drop procedure, we will present our results first in the resummation region, then matched to fixed-order. We then provide an analytic estimate of non-perturbative corrections, extending to the Soft Drop case the analytic results obtained in Ref. [1] for the mMDT. We conclude by providing and discussing our final predictions, including the theory uncertainty bands. These have already been compared to experimental data in [7], where a good agreement was found, especially in the perturbative region.

*Soft Drop*

For a given jet, the Soft Drop procedure first re-clusters the constituents of the jet with the Cambridge/Aachen algorithm [9, 10] into a single jet *j*. Starting from *j*, it then applies the following iterative procedure:

1. undo the last clustering step $j\rightarrow j_1,j_2$, with $p_{t1}>p_{t2}$.
2. stop the procedure if the Soft Drop condition is met:
  1 $$\begin{aligned} \frac{\text {min}(p_{t1},p_{t2})}{p_{t1}+p_{t2}} > z_{\text {cut}}\Big (\frac{\theta _{12}}{R}\Big )^\beta , \end{aligned}$$
  where $z_{\text {cut}}$ and $\beta$ are free parameters, $\theta _{12}^2=\varDelta y_{12}^2+\varDelta \phi _{12}^2$ and *R* the original jet radius.
3. otherwise, set $j=j_1$ and go back to 1, or stop if $j_1$ has no further substructure.

The limit $\beta \rightarrow 0$ corresponds to the mMDT.

*NLL resummation*

We consider the cumulative cross-section for the ratio $m^2/(p_tR)^2$ to be smaller than some value $\rho$, integrated over the $\mathcal{{O}}(\alpha _s^2)$ matrix element for the Born-level production of 2 jets, in a given $p_t$ bin:

2 $$\begin{aligned} \varSigma _{\text {NLL}}(\rho ;p_{t1},p_{t2})=\int _{p_{t1}}^{p_{t2}} \!\!dp_t \sum _i \frac{d\sigma _{\text {jet,LO}}^{(i)}}{dp_t} \frac{e^{-R_i(\rho )-\gamma _ER_i'(\rho )}}{\varGamma (1+R_i'(\rho ))}, \end{aligned}$$

where we have separated contributions from different flavour channels, $R_i'$ is the derivative of $R_i$ wrt $\log (1/\rho )$ and the radiator $R_i$ is given by

3 $$\begin{aligned}&R_i(\rho ) = \frac{C_i}{2\pi \alpha _s\beta _0^2} \Bigg \{ \Bigg [ W(1-\lambda _B)-\frac{W(1-\lambda _c)}{1+\beta }-2W(1-\lambda _1)\nonumber \\&\quad +\frac{2+\beta }{1+\beta }W(1-\lambda _2) \Bigg ] -\frac{\alpha _s K}{2\pi } \Bigg [\log (1-\lambda _B)-\frac{\log (1-\lambda _c)}{1+\beta }\nonumber \\&\quad +\frac{2+\beta }{1+\beta }\log (1-\lambda _2) -2\log (1-\lambda _1) \Bigg ] +\frac{\alpha _s \beta _1}{\beta _0} \Bigg [V(1-\lambda _B)\nonumber \\&\quad -\frac{V(1-\lambda _c)}{1+\beta }-2V(1-\lambda _1) +\frac{2+\beta }{1+\beta }V(1-\lambda _2) \Bigg ] \Bigg \}, \end{aligned}$$

where

4 $$\begin{aligned} \lambda _c&= 2\alpha _s\beta _0\log (1/z_{\text {cut}}), \quad \lambda _\rho = 2\alpha _s\beta _0\log (1/\rho ), \end{aligned}$$

5 $$\begin{aligned} \lambda _1&= \frac{\lambda _\rho +\lambda _B}{2}, \quad \lambda _2 = \frac{\lambda _c+(1+\beta )\lambda _\rho }{2+\beta }, \end{aligned}$$

and $\lambda _B=2\alpha _S\beta _0B_i$ appears due to hard-collinear splittings, and $W(x)=x\log (x)$, $V(x)=\frac{1}{2}\log ^2(x)+\log (x)$.

Note that $\alpha _s$ is calculated using the exact two-loop running coupling, at the scale $p_tR$, and, in order to reach NLL accuracy, it is evaluated in the CMW scheme [11]. Furthermore, compared to the original results [2], the hard-collinear contributions have been expressed as corrections to double-logarithm arguments. In practice, this is equivalent to replacing $P_i(z)\rightarrow (2C_i/z)\varTheta (z<e^{B_i})$. This introduces unwanted NNLL terms but has the advantage to give well-defined and positive resummed distributions which, in turn, makes the matching to fix order easier.

To avoid any potential issue related to the Landau pole, appearing in a region anyway dominated by hadronisation, we have frozen the coupling at a scale $\mu _{\text {fr}}=1$ GeV. Corresponding expressions can be found e.g. in Ref. [12].

*Matching to NLO*

The Soft Drop mass distributions for the dijet processes can be calculated at fixed order at $\mathcal{{O}}(\alpha _s^4)$, i.e. up to NLO accuracy. This is available for example using the NLOJet++ [13, 14] generator to simulate $2\rightarrow 3$ events at LO and NLO. Jets are then clustered with the anti-$k_t$ algorithm [15] as implemented in FastJet-3.2.2 [16, 17]. In what follows, we have used the CT14 PDF set [18].

NLO mass distributions need to be matched to our NLL resummed results. For this, the LO jet mass distribution needs to be separated in flavour channels, while the flavour separation of the NLO jet mass distribution is instead subleading. At $\mathcal{{O}}(\alpha _s^3)$ a jet has at most two constituent and the only case where the flavour is ambiguous is when a jet is made of two quarks (or a quark and an anti-quark of different flavours). We (arbitrarily) treat this as a quark jet, an approximation which is valid at our accuracy. To keep the required flavour information in NLOJet++, we have used the patch introduced in Ref. [19].

To avoid artefacts at large mass, the endpoint of the resummed calculation is matched to the endpoint of the perturbative distribution by replacing

6 $$\begin{aligned} \log \left( \frac{1}{\rho }\right) \rightarrow \log \left( \frac{1}{\rho }-\frac{1}{\rho _{\text {max},i}}+e^{-{B_q}}\right) \end{aligned}$$

in the resummed results [20]. The endpoints of the LO and NLO distributions are found to be (see Appendix B of Ref. [5]) $\rho _{\text {max,LO}} \approx 0.279303$ and $\rho _{\text {max,NLO}} \approx 0.44974$, for $R=0.8$.

Finally, the matching between NLL and NLO results in each $p_t$ bin can be done using log-R matching given by [19]

7 $$\begin{aligned}&\varSigma _{\text {NLL+NLO}}(\rho ) \nonumber \\&\quad = \left[ \sum _i \varSigma _{\text {NLL}}^{(i)} \exp \left( \frac{\varSigma _{\text {LO}}^{(i)}-\varSigma _{\text {NLL,LO}}^{(i)}}{\sigma _{\text {jet,LO}}^{(i)}} \right) \right] \nonumber \\&\qquad \times \exp \left( \frac{\bar{\varSigma }_{\text {NLO}}-\varSigma _{\text {NLL,NLO}}}{\sigma _{\text {jet,LO}}} -\sum _i\frac{(\varSigma _{\text {LO}}^{(i)})^2-(\varSigma _{\text {NLL,LO}}^{(i)})^2}{\sigma _{\text {jet,LO}}^{(i)}\sigma _{\text {jet,LO}}}\right) . \end{aligned}$$

In this expression, $\varSigma _{\text {NLL}}^{(i)}$ is given by Eq. (2), trivially split in flavour channels. $\varSigma _{\text {NLL,LO}}^{(i)}$ and $\varSigma _{\text {NLL,NLO}}$ (summed over flavour channels) are the expansion of $\varSigma _{\text {NLL}}^{(i)}$ to LO ($\mathcal{{O}}(\alpha _s^3)$) and NLO ($\mathcal{{O}}(\alpha _s^4)$), respectively. For the fixed-order part

8 $$\begin{aligned}&\varSigma _{\text {LO}}^{(i)} =-\int _\rho ^1 d\rho '\,\frac{d\sigma _{\text {mass,LO}}^{(i)}}{d\rho '} + \sigma _{\text {jet,NLO}}^{(i)}, \end{aligned}$$

9 $$\begin{aligned}&\bar{\varSigma }_{\text {NLO}} =-\int _\rho ^1 d\rho '\,\frac{d\sigma _{\text {mass,NLO}}}{d\rho '}, \end{aligned}$$

where $d\sigma _{\text {mass,(N)LO}}/d\rho$ denotes the mass distribution at (N)LO as obtained from NLOJet++ and $\sigma _{\text {jet,(N)LO}}$ the (N)LO correction to the inclusive jet cross-section in the $p_t$ bin under consideration. These expressions also require the inclusive jet cross-section, both at LO and NLO, to be split in flavour channels. This is done as for the 3-jet LO distribution above using the flavour-aware NLOJet++ version used in [19]. Alternatively, we have also used the (*R*-)matching scheme given by Eq. (3.28) of [19].

From Eq. (7) it is trivial to obtain differential distributions in bins of $\rho$. Normalised distributions can then be obtained by dividing the result by the NLO inclusive jet cross-section $\sigma _{\text {jet,LO}}+\sigma _{\text {jet,NLO}}$.^2^

**Fig. 1:**
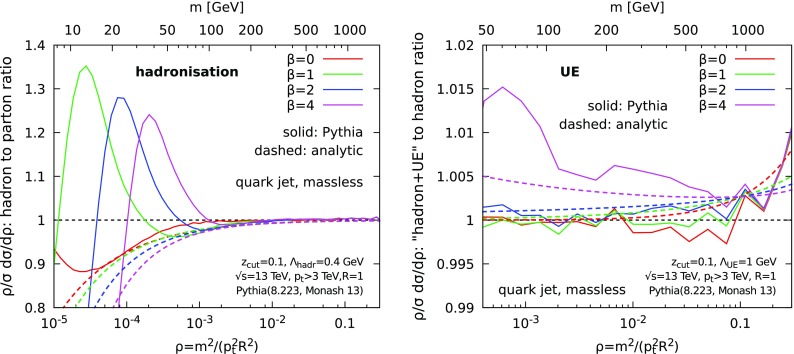
Comparison of our theoretical estimate of hadronisation corrections to what is implemented in a standard Monte-Carlo parton shower, for different values of the angular exponent $\beta$ , $z_{\text {cut}}=0.1$ and $R=0.8$. Quark jets are considered and hadron masses are neglected. Left: hadronisation corrections (i.e. ratio of hadron level to parton level) for $\varLambda _{\text {hadr}}= 0.4$ GeV; right: Underlying Event corrections (i.e. ratio of distributions with and without UE) for $\varLambda _\text {UE}= 1$ GeV

The uncertainties on the distributions come from five sources: renormalisation and factorisation scales, resummation uncertainty, freezing-scale uncertainty and matching uncertainty. The first two are estimated using the 7-point rule [21]. The resummation uncertainties are obtained by varying $\rho$ in Eqs. (2) and (3) between $\rho /2$ and $\rho$, introducing the appropriate correction – $\pm \log (2)R'$ in the exponent in (2) – to maintain NLL accuracy. The freezing scale uncertainty is obtained by considering three values for $\mu _\text {fr}=0.5, 1.0, 1.5$ GeV,^3^ while setting all other scales equal to their central value. The matching uncertainty is estimated by considering both the log-*R* and *R* matching schemes. We take the central value from the central scale choice and the uncertainty from the envelope of the scale variations.

**Fig. 2:**
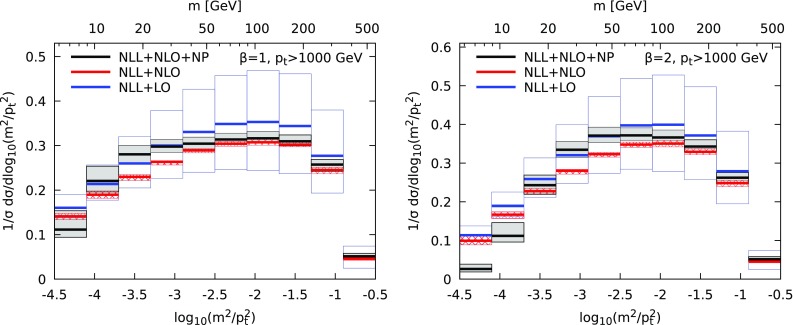
Resummed and match theoretical predictions for the Soft Drop jet mass distribution, for two different values of the angular exponent $\beta =1$ (left) and $\beta =2$ (right), $z_{\text {cut}}=0.1$ and $R=0.8$. The colours correspond to different accuracy of the calculation, as detailed in the legend

*Non-perturbative corrections*

Power corrections induced by non-perturbative (NP) effects can be estimated for Soft Drop using the same approach as the equivalent calculation for mMDT presented in Section 8.3.3 of Ref. [1]. We have to take into account two effects: (i) the mass of the SD jet will be affected by NP corrections, (ii) NP effects can shift the momentum of the subjets and alter the SD condition.

First, the mass shift can be written as (see [22]) $\delta m^2 = C_R\varLambda _{\text {hadr}}p_t R_{\text {eff}}$, where $R_{\text {eff}}$ is the effective jet radius after grooming, i.e. for a mass *m* and subjets passing the Soft Drop condition with a momentum fraction *z*, $R_{\text {eff}}=m/(p_t\sqrt{z(1-z)})$. Following the same steps as in Ref. [1] we obtain^4^

10 $$\begin{aligned} \frac{d\sigma }{dm}\Bigg |_{\text {hadr}}^{(m\text { shift})} = \frac{d\sigma }{dm}\Bigg |_{\text {pert}} \Bigg (1+\frac{C_R\varLambda _{\text {hadr}}}{m}\,\frac{z_{\text {SD}}^{-1/2}-\varDelta _i}{L_{\text {SD}}+B_i}\Bigg ), \end{aligned}$$

with $$z_{\text {SD}}=z_{\text {cut}}^{\frac{2}{2+\beta }}\big (\frac{m}{p_tR}\big )^{\frac{2\beta }{2+\beta }}$$, $$L_{\text {SD}}=\log (1/z_{\text {SD}})$$ and

11 $$\begin{aligned} \varDelta _q=\frac{3\pi }{8}\quad \text { and }\quad \varDelta _q=\frac{(15C_A-6n_fT_R)\pi }{32\,C_A}. \end{aligned}$$

Then, hadronisation will shift the momentum of the softer subjet by an average $\delta p_t=-C_A\varLambda _{\text {hadr}}/R_{\text {eff}}$, where we have taken into account that the softer subjet typically corresponds to a gluon emission. This means that emissions which were perturbatively passing the Soft Drop condition, with $z_{\text {SD}}<z<z_{\text {SD}}-\delta p_t/p_t$, will fail the Soft Drop condition after hadronisation, leading to a reduction of the cross-section

12 $$\begin{aligned} \frac{d\sigma }{dm}\bigg |_{\text {hadr}}^{(p_t\text { shift})} = \frac{d\sigma }{dm}\bigg |_{\text {pert}} \bigg (1-\frac{C_A\varLambda _{\text {hadr}}}{m}\,\frac{z_{\text {SD}}^{-1/2}}{L_{\text {SD}}+B_i}\bigg ). \end{aligned}$$

The final hadronisation correction includes both (10) and (12). Both terms are proportional to $\frac{\varLambda _{\text {hadr}}}{p_t}\big (\frac{p_t}{m}\big )^{\frac{2+2\beta }{2+\beta }}$, which increases with $\beta$ and has the appropriate limits for $\beta \rightarrow \infty$ and $\beta \rightarrow 0$.

A similar calculation can be carried out for the Underlying Event (UE) contamination. In this case we have $\delta p_t=\varLambda _{\text {UE}}\pi R_{\text {eff}}^2$ and $\delta m^2=\frac{1}{2}\varLambda _{\text {UE}}p_tR_{\text {eff}}^4$. Following the same steps as above, we find

13 $$\begin{aligned} \frac{d\sigma }{dm}\Bigg |_{\text {UE}} = \frac{d\sigma }{dm}\Bigg |_{\text {pert}} \Bigg (1+\frac{\varLambda _{\text {UE}} m^2}{p_t^3R^3}\,\frac{z_{\text {SD}}^{-2}(1-f_{m,i})}{L_{\text {SD}}+B_i}\Bigg ), \end{aligned}$$

where the 1 in the numerator corresponds to the $p_t$ shift and the $f_{m,i}$ term corresponds to mass-shift effects, with

14 $$\begin{aligned} f_{m,q}&= \frac{1+3z_{\text {SD}}+2z_{\text {SD}}^2(3L_{\text {SD}}-2)}{4},\nonumber \\ f_{m,g}&= \frac{1+2z_{\text {SD}}+3z_{\text {SD}}^2(2L_{\text {SD}}-1)}{4} + \frac{n_fT_R}{C_A}z_{\text {SD}}(1-z_{\text {SD}}). \end{aligned}$$

This time, both sources of corrections give an effect proportional to $\frac{\varLambda _{\text {UE}}}{p_t}\big (\frac{p_t}{m}\big )^{\frac{2\beta -4}{2+\beta }}$, which increase with $\beta$ and has the expected $\varLambda _{\text {UE}}p_t/m^2$ behaviour in the limit $\beta \rightarrow \infty$.

In Fig. 1, we compare our analytic findings (dashed lines) with the Monte-Carlo simulations, obtained with Pythia 8.223 [23] (Monash 13 [24] tune, solid lines). We consider both hadronisation corrections (left) and UE effects (right), for a range of $\beta$ values. UE effects are seen to be much smaller than hadronisation corrections. In the region where $\varLambda _{\text {hadr,UE}}\ll m \ll p_t$, our analytic calculation captures the main features observed in the simulations, including the increase with $\beta$ and the global trend in $\rho$. At smaller mass, Pythia simulations exhibit a peak in the hadronisation corrections which is beyond the scope of our power-correction calculation.

Even if the above analytic approach to estimating NP effects is helpful for a qualitative understanding, it is unclear how reliable it would be for phenomenology. For example, hadron masses, which are neglected here, would have an additional effect, even at large mass. Thus, the analytic estimates can, at best, be trusted up to a fudge factor and analytic results can not be trusted at small mass (see also [25]).

As for our mMDT calculation [5], for our final predictions, we have therefore decided to estimate NP corrections using different Monte-Carlo simulations: Herwig 6.521 [26] with the tune AUET2 [27], Pythia 6.428 [28] with the Z2 [29] and Perugia 2011 [30, 31] tunes, and Pythia 8.223 [23] with the 4C [32] and Monash 13 [24] tunes. For each Monte-Carlo, we compute the ratio between the full simulation and the parton level. The average result is taken as the average NP correction, and the envelope as the uncertainty which is added in quadrature to the perturbative uncertainty.

*Final predictions and conclusions*

Our final predictions, are presented for $\beta =1$ (left) and $\beta =2$ (right) in Fig 2. To highlight our key observations, we present our final results at NLL matched to NLO and including NP corrections (labelled NLL+NLO+NP), as well as pure perturbative results (NLL+NLO) and results obtained when matching to LO only (NLL+LO). The most striking feature that we observe is that matching to NLO not only affects quite significantly the central value of our prediction, but also significantly reduces the uncertainty across the entire spectrum.

Then, we see that NP corrections remain small over a large part of the spectrum, although they start being sizeable at larger mass when the angular exponent $\beta$ increases. The fact that Soft Drop observables can be computed precisely in perturbative QCD, with small NP corrections, makes them interesting for further phenomenological studies, including other observables like angularities or attempts to extract the strong coupling constant from fits to the data.

Finally, we note that these predictions have recently been compared to experimental results obtained by the ATLAS collaboration in Ref. [7]. A good overall agreement between data and theory is observed, especially in the region where NP corrections are small.

## Footnotes

1 We note that explicit comparisons performed by the ATLAS collaboration indicate that, in the region where perturbation theory is valid, the NNLL contribution is a small correction to NLL and lies within its uncertainties (see Fig. 3 of Ref. [7]).

2 Note that this normalisation procedure gives consistent results when computing the uncertainties on the matched distributions.

3 This remains within our non-perturbative uncertainties (see below).

4 Although, instead of averaging $R_{\text {eff}}$ over *z*, we have kept explicit the *z* dependence of $R_{\text {eff}}$ and averaged the final correction over *z*.
